# Innominate Artery Cannulation for Proximal Aortic Surgery

**DOI:** 10.21470/1678-9741-2022-0045

**Published:** 2023

**Authors:** Bülent Mert, Kamil Boyacioglu, Hakan Sacli, Berk Özkaynak, Ibrahim Kara, Adil Polat

**Affiliations:** 1 Department of Cardiovascular Surgery, Bagcilar Training and Research Hospital, Istanbul, Turkey.; 2 Department of Cardiovascular Surgery, Sakarya Universitesi Tip Fakultesi, Sakarya, Turkey.

**Keywords:** Dissecting Aneurysm, Axillary Artery, Aortic Aneurysm, Brachiocephalic Trunk, Cardiopulmonary Bypass, Catheterization, Neurologic Manifestation

## Abstract

**Introduction:**

The aim of this study was to evaluate the efficacy and safety of innominate artery cannulation strategy with side-graft technique in proximal aortic pathologies.

**Methods:**

A total of 70 patients underwent innominate artery cannulation with a side graft for surgery on the proximal aorta from 2012 to 2020. There were 46 men and 24 women with an average age of 56±13 years. The indications for surgery were type A aortic dissection in 17 patients (24.3%), aortic aneurysm in 52 patients (74.3%), and ascending aorta pseudoaneurysm in one patient (1.4%). The innominate artery was free of disease in all patients. Hypothermic circulatory arrest with antegrade cerebral perfusion was utilized in 60 patients (85.7%). Three patients had previous sternotomy (4.2%). The most common surgical procedure was ascending aorta with hemiarch replacement in 34 patients (48.5%).

**Results:**

The mean cardiac ischemia and cardiopulmonary bypass times were 116+46 minutes and 164+56 minutes, respectively. Mean antegrade cerebral perfusion time was 27+14 minutes. The patients were cooled between 22°C and 30°C during surgery. Thirty-day mortality rate was 7.1% (five patients). One patient (1.4%) had stroke, one patient (1.4%) had temporary neurologic deficit, and eight patients (11.4%) had confusion and agitation that resolved completely in all cases. There was no local complication or arterial injury.

**Conclusion:**

Cannulation of the innominate artery with side graft is safe and effective for both cardiopulmonary bypass and antegrade cerebral perfusion. This technique provides satisfactory neurologic outcomes for proximal aortic surgery.

## INTRODUCTION

Management of the cerebral protection methods in proximal aortic surgery plays a significant role in neurological outcomes. Hypothermic circulatory arrest (HCA) with antegrade cerebral perfusion (ACP) is widely used worldwide to minimize the risk of brain damage during aortic surgery. ACP by cannulation of the right axillary artery provides improved outcome and it has been demonstrated a safe and an effective method^[^1^, ^2^, ^3^, ^4^]^. Nevertheless, this approach has some pitfalls such as brachial plexus injury, seroma, and limb ischemia^[^5^,^6^]^. Innominate artery (IA) cannulation, which was described by Banbury and Cosgrove in 2000^[^7^]^ for ACP, is an alternative strategy to avoid these complications and has gained popularity recently^[^8^, ^9^, ^10^]^. In this study, we evaluated the neurological and overall outcomes in patients undergoing proximal aortic surgery with IA cannulation.

## METHODS

The study was conducted in accordance with principles of the Declaration of Helsinki, and study protocol was approved by Institutional Ethics Committee (No: 2020.09.2.08.135). All patients provided informed consent for data collection. The preoperative, intraoperative, and postoperative data were obtained from the supplemented by surgeons’ report of the operation and hospital records.

### Patient Profile

This retrospective, double-center study includes 70 consecutive patients who underwent IA cannulation with a side graft during proximal aortic surgery from 2012 to 2020. All procedures were performed by median sternotomy. The patients’ baseline demographic characteristics are summarized in Table 1.

**Table 1:** Preoperative patients’ clinical data.

Variables	Results
Age, years	56±13 (19-76)
Sex	
Male	46 (65.7)
Female	24 (34.3)
Hypertension	61 (87.1)
Diabetes mellitus	12 (17.1)
Hyperlipidemia	17 (24.2)
Tobacco use	28 (40)
Chronic obstructive pulmonary disease	16 (22.8)
Renal disease	4 (5.7)
Coronary artery disease	21 (30)
Bicuspid aortic valve	18 (25.7)
Peripheral vascular disease	7 (10)
Cerebrovascular event	5 (7.1)
Redo cardiac surgery	3 (4.2)
Ejection fraction (%)	
≤ 30	0 (0)
31-50	8 (11.4)
> 50	62 (88.5)
Diagnosis	
Type A aortic dissection	17 (24.3)
True aneurysm	52 (74.3)
Pseudoaneurysm	1 (1.4)

Computed tomography angiography was performed in all patients to evaluate the entire aorta and its major branches and iliofemoral arteries. The IA was confirmed to be appropriate for cannulation on computed tomography angiography in all cases. All elective patients older than 40 years old underwent coronary angiography to rule out coronary artery disease.

We performed IA cannulation only in appropriate patients. IA cannulation was not performed if any of the following situations existed: atherosclerosis and/or aneurysmal dilatation of the innominate and type A aortic dissection extending into the IA. Open distal anastomosis was performed in all except 10 cases (85.7%). In these cases, the cross-clamp was not removed due to the sufficient diameter and length of the ascending aorta for distal anastomosis.

### Study Definitions

Preoperative cardiac disease unrelated to the aorta was defined as a history of arrhythmia, previous cardiac surgery, coronary artery disease, valvular disease, or heart failure. Pulmonary disease was defined as forced expiratory volume in 1 second/ forced vital capacity < 70% and/or a history of obstructive or restrictive lung disease. Preoperative renal disease was defined as a creatinine level ≥ 1.2 mg/dl, and hemodialysis dependence was defined as a chronic renal failure. Operative times were defined as follows: cardiac ischemia time was the period from the beginning of circulatory arrest or cross-clamping until clamp removal; cardiopulmonary bypass (CPB) time was the period during which the patient was supported by CPB, not including the ACP time or cerebral circulatory arrest time; ACP time was the duration of circulatory arrest during which the patient received ACP; lastly, circulatory arrest time was the overall time of circulatory arrest without ACP. All of the postoperative neurologic events were classifed into these categories: stroke was defined as any new brain injury evident either clinically or radiographically after the procedure; reversible motor dysfunction in the body was defined as a temporary neurologic deficit (TND) with no focal deficit on computed tomography or magnetic resonance imaging tools; confusion and agitation. Operative mortality was defined as death within 30 days or before hospital discharge. Postoperative renal dysfunction was defined as a hemodialysis requirement or doubling of serum creatinine level. More than 24 hours of intubation postoperatively was considered as prolonged ventilation.

### Operative Technique

**Fig. 1:**
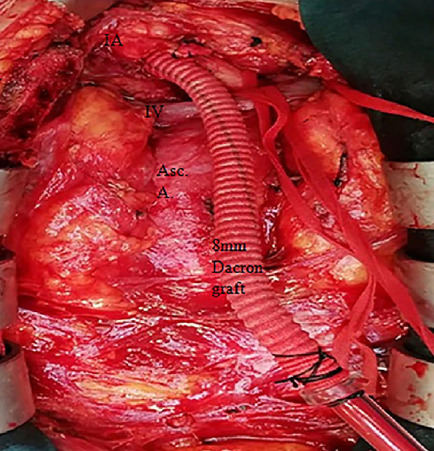
Innominate artery (IA) cannulation with 8-mm side-graft method. The graft is anastomosed to the artery in end-to-side fashion and is cannulated with a 24-F arterial cannula. Asc. A.=ascending aorta; IV=innominate vein.

General anesthesia was used in all patients. Arterial blood pressure was monitored for both arms (radial or brachial arteries). After a standard median sternotomy, the innominate vein was encircled with an umbilical tape and retracted to expose the arcus aorta and its major branches. The IA was exposed and dissected to the bifurcation. An umbilical tape was passed around it to allow caudal retraction. After systemic heparinization (3 mg/kg or 300 IU/kg to achieve activated coagulation time > 480 seconds), a partially side clamp was applied to the distal IA. In cases of small size IA, complete flow occlusion of the IA using two vascular clamps was performed. The right radial artery pressure was measured to evaluate the adequate distal perfusion to the right arm and right cerebral circulation during this manoeuvre. A 10-mm incision was done to the IA. An 8-mm Jotec FlowNit Bioseal® graft (Jotec, Hechingen, Germany) was anastomosed to the artery in end-to-side fashion with a running continuous 5-0 polypropylene suture (Figure 1). The graft was connected to the 24-F arterial line after the side clamp removing and the de-airing. Atriocaval cannulation was used for venous return. CPB was initiated, and the patients were cooled between 22°C and 30°C. Cold blood cardioplegia was given directly into the coronary ostia every 15–20 minutes following an initial retrograde administration. Proximal aortic anastomosis and/or reconstruction was performed initially while the patient was being cooled. Once the target systemic temperature was achieved, ACP began for hemiarch or arcus aortic reconstruction. The proximal IA was occluded using a metal bulldog clamp. Left carotid artery was routinely clamped, but not the left subclavian artery. Ice was packed around the patient’s head. Hydrocortisone and mannitol were also given to avoid cerebral edema, which can occur during and after cooling. While the administration of ACP, cerebral perfusion flow rates were maintained at 10 to 15 mL/kg/min to keep a right radial artery pressure of 50 to 70 mmHg.

**Fig. 2:**
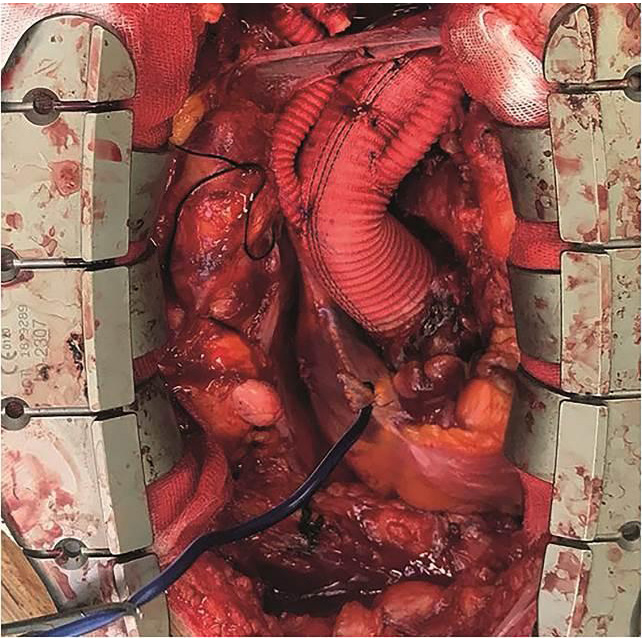
Using the innominate artery cannulation graft for proximal arch repair. In this patient, who underwent ascending aorta and proximal arcus replacement (innominate artery and left common carotid artery), the graft was anastomosed to the aortic graft for proximal arch repair after decannulation of the innominate artery.

**Fig. 3:**
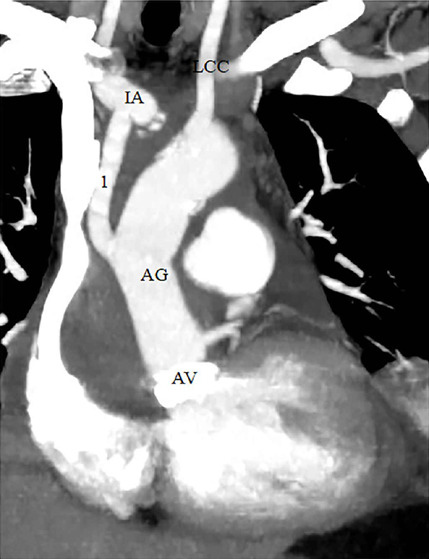
Using the innominate artery (IA) cannulation graft for proximal arch repair. Computed tomography angiography showing that the graft used for IA cannulation was anastomosed between the ascending aortic graft (AG) to IA in a patient who underwent Bentall and proximal arcus repair operation. AV=aortic valve; LCC=left common carotid artery; 1=graft to IA.

When the distal anastomosis was complete, ACP was discontinued. After protamine administration, the 8-mm graft was cut and oversewn with a double 5-0 polypropylene running suture. The total body and cerebral perfusion was maintained by IA during the entire procedure, except for the aortic arch repair. In aortic arch repair, extracorporeal circulation was reinstituted in an antegrade fashion through the ascending aortic graft via new aortic cannula for body and cerebral perfusion, and then the IA graft was anastomosed to the ascending aortic graft (Figures 2 and 3).

In just one patient who had huge ascending aorta pseudoaneurysm due to the aortotomy incision, we performed femoral artery and vein cannulation to establish CPB before sternotomy. Due to severe adhesion around the distal part of the ascending aorta, we used open technique and we performed IA for ACP during the aortic repair with porcine pericardial patch (Biointegral Surgical No-React® Patch, Toronto, Canada).

### Statistical Analysis

Statistical analyses were performed using the statistical software SPSS 15.0 for Windows (SPSS Inc., Chicago, United States of America). Data are expressed as mean ± standard deviation for continuous variables and as counts with percentages for categorical variables.

## RESULTS

IA cannulation using 8-mm Dacron graft was successful in all patients. Details of the surgical procedures and operative times are presented in Table 2. There were no local complications, and there was no need to change cannulation site due to malperfusion or arterial injury in any of the patients. The postoperative outcomes of the patients are summarized in Table 3.

**Table 2:** Operative procedures and times.

Surgical procedures	Results
Aortic procedures	
Ascending aortic replacement only	16 (22.8)
Ascending and hemiarch replacement	34 (48.5)
Bentall	18 (25.7)
Valve sparing root replacement	1 (1.4)
Proximal arch repair	9 (12,8)
Total arch repair	0 (0)
Aortoplasty with patch	1 (1.4)
Other surgical procedures	
Aortic valve replacement	17 (24.2)
Aortic valve repair	7 (10)
Coronary artery bypass grafting	9 (12.8)
Tricuspid, mitral repair	4 (5.7)
Others	4 (5.7)
Noncardiac surgical procedures	
Cross-femoral bypass	1 (1.4)
Intraoperative times (min)	
Cardiopulmonary bypass time (n = 70)	164 (65-326)
Cardiac ischemia time (n = 70)	116 (33-178)
Antegrade cerebral perfusion time (n = 60)	27 (11-74)

**Table 3:** Postoperative outcomes.

Variables	Results
30-day mortality	5 (7.1)
Neurologic outcomes	
Stroke	1 (1.4)
TND	1 (1.4)
Confusion and agitation	8 (11.4)
Cardiac rhythm disturbances	
Atrial fibrillation	15 (21.4)
Permanent pacemaker	1 (1.4)
Reoperation for bleeding	7 (10)
Renal failure requiring hemodialysis	3 (4.2)
Deep sternal wound infection	4 (5.7)
Tracheostomy	2 (2.8)
Mechanical ventilation > 24 hours	17 (24.2)
Intensive care unit stay (days)	6.5 (2-70)

TND=temporary neurologic deficit

The postoperative 30-day mortality rate was 7.1% (five patients). Two patients sufered from type A aortic dissection and required emergency operation. Ascending and hemiarch replacement was done at the first patient who had had coronary artery bypass grafting, but he died on postoperative day one due to myocardial infarction. The second patient sufered from cerebrovascular event before the surgery. Ascending and hemiarch replacement was performed on this patient. He died at the operative day because of low cardiac output syndrome. The other three patients underwent elective operations due to aortic aneurysm. The first two patients died due to low cardiac output syndrome. Aortic root, hemiarch replacement, and coronary artery bypass grafting were done on one of them, and ascending aorta and triple coronary artery bypass grafting were done on the other patient. These patients died at early postoperative period. The last patient, who had chronic obstructive pulmonary and chronic renal diseases, died on postoperative day 26 due to renal failure and infection. The patient had exhibited multiorgan failure before death.

One patient experienced postoperative stroke (1.4%). This patient underwent emergency operation due to type A aortic dissection. He had apparent confusion with right hemiparesis before surgery. Aortic root replacement was performed in this patient with short-time unilateral ACP (24 minutes). In the intensive care unit, follow-up tracheostomy and percutaneous endoscopic gastrostomy were performed postoperatively. The left-sided large hemispheric was intact on computed tomography and magnetic resonance imaging during the follow-up. He was transferred to another center after two months and died on the 6th month. One patient (1.4%) presented TND with complete resolution before being discharged. A state of postoperative confusion and agitation was observed in eight patients (11.4%). These manifestations resolved within various times in all of the patients (two days-one week).

## DISCUSSION

Neurologic outcomes are one of the key points that determine the success of proximal aortic surgery. Therefore, modifed surgical techniques and cerebral protection methods for reducing the risk of neurological complications have been established in the history of aortic surgery. Current, HCA with ACP is a popular approach for cerebral protection during aortic surgery. Although right axillary artery cannulation is chosen widely around the world, the frequency of use of IA cannulation increased recently^[^11^]^. In this study, we performed IA cannulation with 8-mm side graft in aortic aneurysm and dissection. According to our results, IA cannulation with side graft is simple and safe with low neurologic morbidity rate. We believe that this method may be preferred in certain patients safely.

The strategy of ACP with moderate hypothermia provides brain protection during aortic surgery and produces better neurological outcomes than cannulating the femoral artery with only deep HCA^[^2^,^3^]^. Today, many surgeons prefer right axillary artery cannulation for ACP in elective and emergency cases. Although axillary artery cannulation is usually well tolerated, various technical challenges and adverse consequences may be associated with this method^[^6^,^12^]^. Firstly, it requires a separate incision and after the cannulation arterial injury, brachial plexus injuries, arm ischemia, inadequate cardiopulmonary flow, malperfusion, and seroma formation may occur^[^12^, ^13^, ^14^]^. Also, blood loss from axillary artery incision during surgery may contribute to further coagulopathy at the postoperative period^[^15^]^. But, axillary artery cannulation via side graft is suggested to reduce the risk of complications^[^6^]^.

Another option for ACP in aortic surgery is IA cannulation. IA cannulation possesses several advantages: since the additional incision for exposure is not necessary, the operation time may be shorter in similar aortic pathology; blood loss and possible kinking of the cannula can be minimized during surgery because the cannulation site is always under the surgeon’s view; the risk of brachial plexus injuries and arm ischemia associated with axillary artery cannulation are avoided; because of the IA diameter, total CPB flow can easily performed without the need for higher pressure; ACP pressure can be measured via right radial artery cannula; in obese patients, the cannulation technique is easier; IA cannulation provides antegrade cerebral flow directly, so the risk of retrograde cerebral embolism is eliminated from the thoracoabdominal aorta, unlike the femoral artery cannulation; finally, in the aortic arch repair, the side graft used for cannulation to IA may be anastomosed to the aortic graft, and thus, proximal aortic arch can be replaced after the termination of ACP. We performed all proximal arch replacements (IA only or IA and left common carotid artery) with this method (nine patients). Two different techniques have been described for IA cannulation. Direct cannulation with different size of arterial cannula or side-graft anastomosis can be chosen for CPB and ACP^[^8^, ^9^, ^10^, ^11^,^15^, ^16^, ^17^, ^18^, ^19^]^. Since 2012, we have used IA cannulation in proximal aortic repairs with side-graft technique in all appropriate patients for both CPB and ACP. We did not encounter any local complications in any of our patients in terms of cannulation site.

IA cannulation in proximal aortic surgery without regard of cannulation technique have provided excellent outcomes in other surgical teams. In a series with 55 patients undergoing aortic replacement with IA cannulation by side-graft technique mainly for aneurysmal disease, 3.6% hospital mortality and 1.8% transient neurologic dysfunction were reported. The stroke rate was zero in the same sample^[^16^]^. Another group reported a sample of 68 patients (including aortic dissection and aneurysmal disease) undergoing proximal aortic surgery where a side graft was sewn to the IA. Their 30-day mortality rate was 1.5%, and stroke rate was 4.4% (three patients) (two of whom had a partial recovery). Moreover, 10.3% of patients have developed temporary postoperative confusion that resolved successfully in all cases^[^20^]^. In a larger sample, including 263 patients, the same group demonstrated their new results with the same technique. The outcomes of the study were quite satisfactory; so that the operative mortality rate was 4.9%, and permanent stroke rate was 1.9%^[^9^]^. The other group who used a side-graft technique to cannulate the IA in 46 patients (38 of them were aortic dissection) reported 30-day mortality, stroke, and TND rates of 6.5%, 0%, and 10.9%, respectively^[^18^]^. Postoperative mortality, permanent stroke, and temporary postoperative confusion rates were reported as 2.3-6.25%, 0-3%, and 7.8-15.6%, respectively, in other groups that make IA cannulation using the side-graft technique for proximal aortic surgery^[^15^,^21^]^. In our 70 patients’ series (17 type A aortic dissection, 53 aneurysmal disease), we had five deaths, and 30-day mortality rate was 7.1%. When we view the neurological events in our group, stroke, TND, and postoperative cognitive dysfunction rates were 1.4%, 1.4%, and 11.4%, respectively. We believe that our results are acceptable and comparable with the other groups.

Direct IA cannulation has also similar results to side-graft technique. In a study with 68 patients where 22-F or 24-F size wire-reinforced flexible short-tip cannula was used for CPB and ACP, no neurologic complications were noted and the mortality rate was 2.9%^[^17^]^. Another group who used the same size cannulas with the same method for direct IA cannulation in 54 patients reported stroke, temporary cognitive dysfunction, and hospital mortality rates of 1.8%, 9.2%, and 3.7%, respectively^[^22^]^. These large arterial cannulas are usually used for central aortic cannulation for CPB. This method has the potential to damage the back wall of the artery due to the IA diameter. Although the authors did not report any problems related to these cannulas, the requirement of rerouting the tip of the cannula may injure the IA. One study which used smaller size cannula (14 F) in 50 elective cases showed the following results with 2% stroke rate, 9% delirium rate, and 2% mortality rate^[^8^]^. The 9-F arterial cannula was used in 100 selected patients for ACP by another group. They reported their experiences with stroke and mortality rates as 1% and 1%, respectively^[^10^]^. These two groups used the direct IA cannulation exclusively with ACP, not the whole-body perfusion. This technique includes three steps to achieve the CPB and ACP: cannulating the aorta, cannulating the IA, and lastly, cannulating the graft. Thus, this three-step procedure can be time consuming during surgery. Even though these studies had excellent outcomes for neurologic events and mortality, they do not involve any complex arch pathology or emergency surgery, like acute aortic dissection. The direct IA cannulation may cause the dissection of the artery and narrowing of the IA after decannulation and typing the purse strings^[^18^]^. Moreover, direct cannulation method may lead the sandblasting effect induced by the turbulent flow that could predispose to embolic complications^[^23^]^.

Lastly, in studies comparing right axillary artery with IA cannulation, similar neurologic and mortality outcomes were demonstrated in the proximal aortic surgery^[^5^,^11^,^15^]^. In a comprehensive study, in which was performed propensity score analysis of right axillary (515 patients) and IA (376 patients) cannulation, during elective aortic surgery, both cannulation sites (both via side graft) have been shown to give excellent results and are interchangeable^[^24^]^. Also, it is emphasized that IA cannulation may provide shorter operation times compared with axillary artery cannulation^[^5^,^11^]^.

### Limitations

These operations were performed in two different medical centers by different surgeons with the same method in various indications. This was a non-randomized, observational study including exclusively patients who underwent IA cannulation. No comparison was made with other cannulation site options.

## CONCLUSION

In conclusion, we believe that IA cannulation with side graft is a simple, safe, and effective technique to establish both CPB and ACP for proximal aortic pathologies without any regard to indication for surgery in appropriate patients. Cannulation of IA with side graft provides satisfactory neurologic outcomes, therefore it is an eligible option for CPB and ACP in aortic surgery.
